# Preface to the Special Issue ‘Heavy Metals in Mushrooms’

**DOI:** 10.3390/jof9121163

**Published:** 2023-12-03

**Authors:** Ivan Širić, Miha Humar

**Affiliations:** 1 Faculty of Agriculture, University of Zagreb, Svetosimunska 25, 10000 Zagreb, Croatia; 2 Biotechnical Faculty, University of Ljubljana, Jamnikarjeva 101, 1000 Ljubljana, Slovenia; miha.humar@bf.uni-lj.si

Population growth, intensive industrialization and urbanization have led to environmental pollution, especially soil and water pollution. The increasing presence of environmental pollutants, particularly heavy metals, has become an alarming problem. Heavy metals in the environment are often carcinogenic, mutagenic, neurotoxic and immunogenic. Thus, they enter the food chain, where they bioaccumulate and remain in the human body for a long time. According to the World Health Organization, lead poisoning causes neurological damage in humans, which manifests itself as a decline in intelligence, short-term memory loss, and problems with learning and movement coordination. Arsenic poisoning causes cardiovascular problems associated with skin cancer and other skin diseases, peripheral neuropathies and kidney damage. Cadmium also accumulates in the kidneys and lungs, and mercury damages the nervous system, causes uncontrolled tremors, muscle damage, partial vision loss and deformities in children. Due to the increasing presence of heavy metals and toxic elements in the environment, numerous scientific studies are being conducted worldwide to preserve and protect the environment. One of the first steps in solving the problem of environmental pollution is to monitor the presence and concentration of pollutants, especially heavy metals. To date, the study of pollutant concentrations in the environment has revealed that saprotrophic and ectomycorrhizal fungal species have a high sensitivity to the contamination of substrates and water with heavy metals and metalloids. The accumulation of Fe, Zn, Cu, Ni, Cr, Pb, Cd and Hg in saprophytic and ectomycorrhizal fungi is a complex phenomenon, which depends on numerous external factors, mechanisms within the fungi, their interactions, and genetic characteristics of the species. Fungal mycelium can accumulate in all heavy metals in a much higher concentration than the substrate on which it develops and lives. The density and depth of mycelium, which lives in the soil for several months or years, influence the content of heavy metals in the fruiting bodies of fungi. In addition, various ecological factors and soil properties (pH and organic matter) can influence the concentration of heavy metals in fungi. 

The first article of this Special Issue addresses the molecular tolerance mechanism of *Paraisaria dubia* to Zn^2+^ stress and the possible application of *Paraisaria dubia* in the bioremediation of heavy metal pollution [1]. The second article analyzes the ectomycorrhizal communities in a former uranium mine and communities under experimental conditions to improve plants’ tolerance to certain abiotic conditions [2]. The third article deals with the possibility of using *Yarrowia lipolytica* to remove heavy metals from woods treated with chromated copper arsenate (CCA) [3]. The fourth article describes a risk assessment of total mercury content in edible wild mushrooms in Slovakia, while the fifth article evaluates the concentration of six heavy metals in two edible oyster mushrooms [4,5]. Furthermore, the sixth article examines the mycomedial ability of *Agaricus bisporus* to grow on compost mixed with flotation residues [6]. The seventh article highlights the resistance of white and brown rot fungi to copper in the microcosm of copper/azole-treated wood [7]. The eighth article deals with the health risk assessment of cadmium accumulation in three fungal species in central and coastal Croatia [8]. In contrast, the ninth article underscores the mechanisms of cadmium bioconcentration using *Boletus griseus* and potential bioremediation fungi for alleviating cadmium contamination [9]. The tenth article investigates the exposure to essential and toxic elements upon the consumption of five edible wild mushroom species collected in southern Spain and northern Morocco [10]. The eleventh article reports on a spatial assessment of the concentration of eight toxic elements in *Agaricus bisporus* collected from local markets in India [11]. The twelfth article describes the synergy between agriculture and waste management, using sewage sludge as casing material for the cultivation of mushrooms (*Agaricus bisporus*) [12].

In conclusion, a global heavy metal monitoring program should be established to determine heavy metal concentrations for certain and commonly consumed saprophytic and ectomycorrhizal fungal species from the genera Agaricus, Boletus, Leccinum, Lactarius, Macrolepiota, Suillus, Tricholoma, etc. In addition, such a program should be intensified near significant sources of heavy metal emissions, such as smelters, power plants and major roads. Furthermore, there is a need for constant monitoring and controlling the levels of metals and metalloids in human diet, both in foods of plant and animal origin and in food supplements; in this case, it is edible wild mushrooms, whose availability on the market and general popularity are constantly increasing.
